# Identification of factors that impact recurrence in patients with borderline ovarian tumors

**DOI:** 10.1186/s13048-017-0316-5

**Published:** 2017-04-04

**Authors:** Xi Chen, Chenyan Fang, Tao Zhu, Ping Zhang, Aijun Yu, Shihua Wang

**Affiliations:** 1 Department of Gynecologic Oncology, Zhejiang Cancer Hospital, 1 Banshan East Road, Hangzhou, Zhejiang 310022 China; 2 Department of Cancer Biology, Wake Forest School of Medicine, Winston Salem, NC 27157 USA

**Keywords:** Borderline ovarian tumor, Survival, Recurrence, Fertility-preserving surgery, Lymphadenectomy

## Abstract

**Background:**

The lack of consensus around best practices for management of borderline ovarian tumors (BOT) is, in part, to the lack of available data and of clarity in interpreting relationships among various factors that impact outcomes. The objective of this study was to identify clinicopathological factors that impact prognosis of patients with borderline ovarian tumors (BOT) and to address features of this disease with the objective of providing clarity in decision making around management of BOT.

**Results:**

A total of 178 BOT patients were included in this study, with a median age of 43 years and a median follow-up time of 37 months. Thirty-two (18.0%) recurrences and 5 (2.8%) deaths were observed in this study group. Multivariate analysis showed that fertility-preserving surgery (*P* = 0.0223 for bilateral cystectomy) and invasive implants (*P* = 0.0030) were significantly associated with worse PFS, whereas lymphadenectomy (*P* = 0.0129) was related to improved PFS. No factors were found to be associated with OS due to the limited number of deaths. In addition, patients with serous BOT more commonly had abnormal levels of CA125, while patients with mucinous BOT more commonly had abnormal levels of CEA. Patients with abnormal levels of CA125, or CA19-9, or HE4 had significantly larger tumor sizes.

**Conclusions:**

Our study reveals the impact of certain types of fertility-preserving surgery, lymphadenectomy and invasive implants on PFS of BOT patients. Blood cancer markers may be associated with histology and size of BOT. Our findings may assist in selection of optimum treatment for BOT patients.

**Electronic supplementary material:**

The online version of this article (doi:10.1186/s13048-017-0316-5) contains supplementary material, which is available to authorized users.

## Background

Borderline ovarian tumors (BOT) account for 15–20% of all ovarian tumors [1]. Compared to invasive epithelial ovarian cancers, BOT occurs more commonly at a younger age, during the time of optimum fertility [2]. BOT patients have a good prognosis with 5- and 10-year survival rates of 95 and 92.8% respectively, and recurrence rates of 5–8% [3–5]. Complete staging is currently the standard surgery treatment for BOT patients. However, the manner and extent of management of these patients remains a subject of debate. Further clinical investigation is needed to achieve clarity concerning which types of fertility-preserving surgery are safer for young patients who desire to retain fertility, whether there is a need to remove retroperitoneal lymph nodes, and whether it is necessary to use adjuvant chemotherapy after surgery.

The objective of the present study was to determine the impact of a number of clinicopathological factors on recurrence and survival of BOT patients, and to address features related to this disease.

## Methods

BOT patients treated between January 1996 and December 2015 were identified from medical records of our hospital. BOT were confirmed by pathological diagnoses on surgical specimens. Pathological staging was performed according to the criteria of the International Federation of Gynecology and Obstetrics (FIGO) 2014. Due to the retrospective nature of the study, informed consent was waived by the Medical Ethics Committee of our hospital.

Surgical management was based on extent of disease, patient age, and patient’s desire to preserve fertility. Surgical procedures were classified as radical or fertility-preserving. Removal of both ovaries was classified as radical. The complete staging procedure consisted of total hysterectomy and bilateral accessory resection with or without removal of lymph nodes, resection of the greater omentum below the transverse colon, multiple abdominal biopsies, and peritoneal lavage of exfoliated cells. Fertility-preserving surgery retained the uterus and adnexa at one or both sides. Three types of fertility-preserving surgery were performed: unilateral salpingo-oophorectomy (USO), unilateral salpingo-oophorectomy plus contralateral cystectomy (USO + CC) and bilateral cystectomy (BC). Laparoscopic surgery was selected by patients. All patients that underwent laparoscopic or open surgery had complete staging. Lymphadenectomy was performed based on intraoperative finding of enlarged lymph nodes and/or disseminated foci. Chemotherapy was recommended for all BOT patients with lymph node metastasis, or invasive implant, or at stage III/IV. Follow-up with patients occurred once every 3 months in the first 2 years and every 6 months thereafter. At the time of follow-up, patients received routine gynecological examination, testing for cancer markers and B ultrasound. If cancer biomarkers and/or B ultrasound were abnormal, then patients would be examined by CT.

Recurrence was diagnosed by elevated tumor markers plus imaging diagnosis of ovarian or pelvic mass. Progression-free survival (PFS) was defined as the time from the date of primary surgery to detection of first recurrence or the last follow-up. Overall survival (OS) was defined as the time from the date of primary surgery to BOT-specific death or the last follow-up.

### Statistical analysis

All statistical analyses were performed using SAS 9.3 (SAS Institute Inc, Cary, NC). Chi-square was used to examine categorical data. *T*-test was applied to continuous data of two groups. ANOVA with post-hoc Tukey test was applied for continuous data over two groups. Univariate and multivariate Cox regression models were used to determine the effect of clinicopathological factors on PFS and OS, and results were presented as hazard ratios (HR). The proportionality assumption was checked by adding a covariate created from an interaction of the predictor and the recurrence time in the model. A collinearity of variables used in the final model was examined by a Chi-square test. The Kaplan-Meier method was also used for analysis of impact of individual variables on PFS. A *P* value of < 0.05 was considered to be statistically significant.

## Results

A total of 249 BOT patients were identified. Seventy-one were excluded due to death from other diseases unrelated to BOT (2 cases), or concurrent presence of localized ovarian cancer (46 cases), cervical cancer (12 cases), colorectal cancer (7 cases), and intraperitoneal pseudomyxoma (4 cases). The remaining 178 cases were included in this study and clinicopathological features corresponding to these cases are presented in Table 1. Patient ages ranged from 15 to 87 years with a median age of 43 years. Among them, 90 (50.6%) patients had preoperative CA125 ≥ 35 U/ml; 50 (28.1%) patients had CA19-9 ≥ 35 U/ml; 28 (15.8%) patients had CEA ≥ 3.4 U/ml; and 35 (19.7%) patients had HE4 ≥ 105 U/ml.

**Table 1:** Demographic and clinical features of BOT patients

Variables		N (%)
Age (range 15–87 years)	≤40	78 (44.1)
	>40	99 (55.9)
Multiparous		30 (16.9)
Nulliparous		148 (83.1)
CA199	<35 U/ml	117 (65.7)
	≥35 U/ml	50 (28.1)
	N/A	3 (1.7)
CA125	<35 U/ml	78 (43.8)
	≥35 U/ml	90 (50.6)
	N/A	10 (5.6)
CEA	<3.4 U/ml	138 (77.5)
	≥3.4 U/ml	28 (15.8)
	N/A	12 (6.7)
HE4	<105 U/ml	131 (73.6)
	≥105 U/ml	35 (19.7)
	N/A	12 (6.7)
Fertility-preserving		68 (38.4)
USO		33 (18.6)
USO + CC		14 (7.9)
BC		21 (11.9)
Radical surgery		109 (61.6)
Lymphadenectomy	Yes	99 (56.2%)
	No	77 (43.8%)
Restaging	Yes	60 (33.7)
	No	116 (65.2)
	N/A	2 (1.1)
Rupture	Yes	30 (16.9)
	No	66 (36.3)
	N/A	84 (46.1)
Ascites	Positive	19 (10.7)
	Negative	107 (60.1)
	N/A	52 (29.2)
Surgery approach	Open	166 (93.2)
	Laparoscopic	10 (5.6)
	Laparoscopic to open	2 (1.2)
Chemotherapy	Yes	23 (12.9)
	No	157 (87.1)
Recurrence		32 (18.0)
Death		5 (2.8)

*N/A*, data not available, *USO* unilateral salpingo-oophorectom, *USO + CC* unilateral salpingo-oophorectomy plus contralateral cystectomy, *BC* bilateral cystectomy

There were 108 patients (61.6%) who underwent radical surgery. Sixty-eight (38.4%) patients underwent fertility-preserving surgery, which included 33 (18.6%) USO, 14 (7.9%) USO + CC, and 21 (11.9%) BC. The majority of patients (93.2%) were operated on using open surgery. Laparoscopic surgery was performed in 12 patients (6.8%). Two (1.2%) of them were converted to open surgery due to intraoperative bleeding caused by injury to iliac blood vessels. Lymphadenectomy was performed in 99 patients (56.2%) to remove pelvic lymph nodes and in 36 patients (20.2%) to remove para-aortic lymph nodes. Patients’ clinicopathological factors that significantly correlated with lymphadenectomy and positive pelvic lymph node metastasis are presented in Additional file 1: Tables S1 and S2, respectively. Sixty patients (33.7%) underwent restaging. Ascites of 19 patients (10.7%) were identified with positive tumor cells. Rupture occurred in 30 patients (16.9%) during surgery. A total of 23 patients (12.9%) underwent adjuvant chemotherapy after initial surgery (Table 1). The clinicopathological factors significantly related to chemotherapy are presented in Additional file 1: Table S3.

Pathological information corresponding to BOT is listed in Table 2. The median tumor diameter was 10 cm (range 2–50 cm). Among these tumors, 84 (48.2%) localized to the left, 63 (35.4%) localized to the right and 31 (17.4%) localized bilaterally. Among 115 patients (64.6%) with stage I disease, 76 patients (42.7%) were classified as stage Ia, 13 (7.3%) as stage Ib, and 26 (14.6%) as stage Ic. Fourteen patients (7.8%) had stage II disease, and 27 patients (15.2%) had stage III disease. The histology of BOT included 71 (39.9%) serous tumor, 80 (44.9%) mucinous tumor and 18 (10.1%) endometrioid tumors. Of the serous BOT cases, 20 (28.2%) had micropapillary lesions and 11 (15.5%) had microinvasion lesions. Twenty (25.0%) mucinous BOT had intraepithelial neoplasia. Of all patients, 8 (4.5%) had extraovarian invasive implants. The median number of pelvic lymph nodes removed was 9 (range, 1–50) and the median number of harvested para-aortic lymph nodes was 3 (range, 1–6). Fifteen patients (15.2%) had positive pelvic lymph node metastasis, and six patients (16.7%) had positive para-aortic lymph node metastasis.

**Table 2:** Pathological features of BOT

Variables		N (%)
Location	Left	84 (48.2)
	Right	63 (35.4)
	Bilateral	31 (17.4)
Diameter	≥10 cm	78 (43.8)
	<10 cm	74 (41.6)
	N/A	26 (14.6)
Histology	Serous	71 (39.9)
	Mucinous	80 (44.9)
	Endometrioid	18 (10.1)
	N/A	9 (5.1)
Stage	Ia	76 (42.7)
	Ib	13 (7.3)
	Ic	26 (14.6)
	II	14 (7.8)
	III	27 (15.2)
	N/A	22 (12.4)
Micropapillary^a^	Yes	20 (28.2)
Microinvasion^a^	Yes	11 (15.5)
Intraepithelial neoplasia^b^	Yes	20 (25.0)
Pelvic lymph node	Positive	15 (15.2)
	Negative	84 (84.8)
Para-aortic lymph node	Positive	6 (16.7)
	Negative	30 (83.3)
Invasive implants	Yes	8 (4.5)
Residue		2 (1.1)
		176 (98.9)

*N/A* data not available; ^a^% of serous tumors; ^b^% of mucinous tumor

The median follow-up time was 37 months (range, 11–180 months). At the time of last follow-up, 32 patients (18.0%) had recurrences and 5 (2.8%) of them died of the disease after surgery; two patients (1.1%) were lost to follow-up. Detailed information corresponding to recurrence sites for these patients is presented in Additional file 1: Table S4. These recurrent patients were treated by cytoreductive or staging surgery with or without chemotherapy, or fertility preservation staging surgery.

**Fig. 1:**
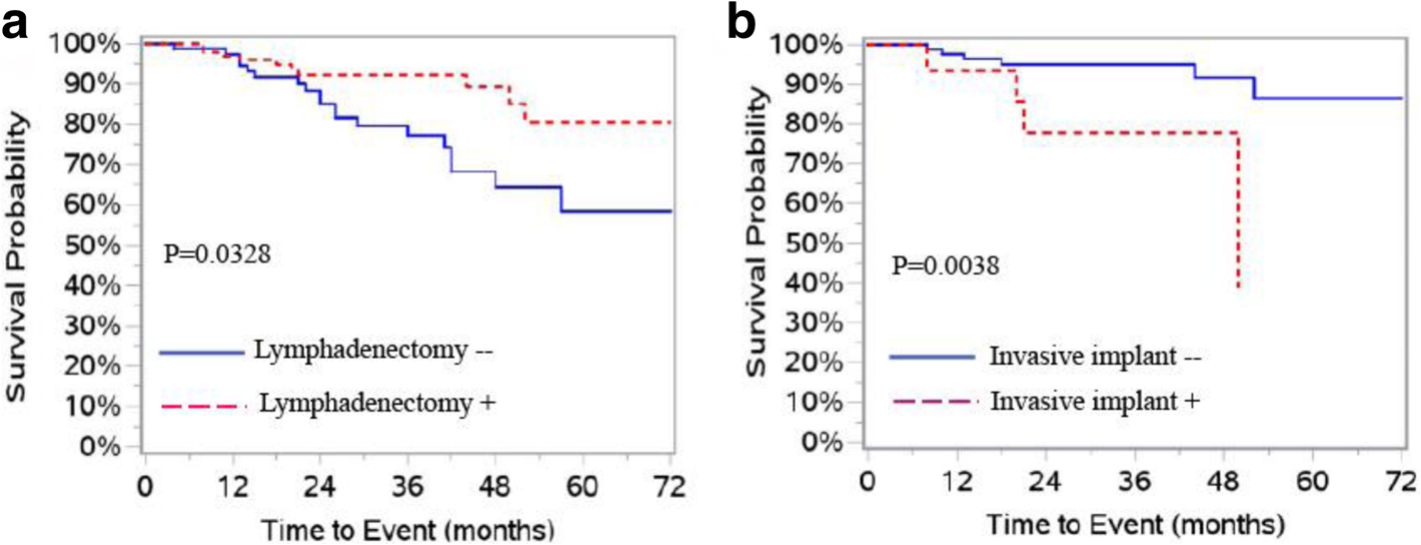
PFS curves in patients with BOT. **a** PFS by lymphadenectomy. **b** PFS by invasive implants

Univariate Cox regression analysis showed that these variables were significantly associated with PFS: tumor diameter (*P* = 0.0076), mucinous histology (*P* = 0.0375), lymphadenectomy (*P* = 0.0328), positive pelvic lymph node metastasis (*P* = 0.0246), para-aortic lymph node metastasis (*P* = 0.0137), tumor stages (*P* = 0.0295), invasive implant (*P* = 0.0038), fertility preserving surgery (*P* = 0.0007 for BC, and *P* = 0.0003 for USO + CC) and adjuvant chemotherapy (*P* = 0.0164). Survival curves by lymphadenectomy and invasive implants are displayed in Fig. 1. The recurrence outcomes categorized with above clinicopathological variables are presented in Additional file 1: Table S5.

A multivariate Cox regression model was built after controlling for tumor histology and stages. Our results showed a significant negative correlation between fertility-preserving surgery and invasive implants to PFS (*P* = 0.0223 for BC and *P* = 0.0030 for invasive implants). Lymphadenectomy was significantly associated with improved PFS (*P* = 0.0129) (Table 3).

**Table 3:** Univariate and multivariate analysis of progression-free survival

Variables		Univariate		*P*	Multivariate^a^		*P*
		HR	95% CI		HR	95% CI	
Multiparous	Yes	0.32	0.15–0.67	0.024			
Nulliparous	No	1					
Diameter	≥10	0.26	0.09–0.70	0.0076			
	<10	1					
Histology	Serous	1					
	Mucinous	0.41	0.18–0.95	0.0375			
	Endometrioid	0.64	0.19–2.19	0.4808			
Micropapillary	Yes	3.88	1.76–8.52	0.0008			
	No	1					
Stage	II & III	2.55	1.10–5.91	0.0295			
	I	1					
Fertility preserving surgery							
USO		1.6	0.59–4.33	0.3553	1.26	0.37–4.08	0.7395
USO + CC		5.74	2.10–15.67	0.0007	2.72	0.65–11.38	0.1719
BC		5.89	2.25–14.42	0.0003	3.95	1.22–12.85	0.0223
Radical surgery		1					
Invasive implant	Yes	4.87	1.67–14.20	0.0038	10.38	2.21–48.69	0.0030
	No	1					
Lymphadenectomy	Yes	0.44	0.21–0.94	0.0328	0.26	0.09–0.75	0.0129
	No	1					
Pelvic lymph node metastasis	Yes	4.34	1.21–15.67	0.0246			
	No	1					
Para-aortic lymph node metastasis	Yes	17.34	1.80–167.50	0.0137			
	No	1					
Chemotherapy	Yes	2.72	1.20–6.19	0.0164			
	No						
Restaging	Yes	9.8	3.96–23.78	<0.0001			
	No						

*USO* unilateral salpingo-oophorectom, *USO + CC* unilateral salpingo-oophorectomy plus contralateral cystectomy, *BC* bilateral cystectomy

^a^Multivariate model was built after controlling for tumor histology and stage

Univariate Cox regression analyses were performed to determine effects of clinicopathological variables on OS. No factors were found to be significantly associated with OS in both models.

The relationship between histology of BOT and blood cancer markers CA19-9, CA125, CEA, and HE4 was determined (Table 4). Our results showed that patients with serous BOT were more likely to have abnormal CA125 (*P* = 0.025), and patients with mucinous BOT were more likely to have abnormal CEA levels (*P* = 0.0005).

**Table 4:** Relationship between blood cancer markers and histology of BOT

Markers		Histology			*P* ^*^
		Serous	Mucinous	Endometrioid	
CA199	<35 U/ml	52 (46.4)	47 (42.0)	13 (11.6)	0.2959
	≥35 U/ml	17 (24.7)	27 (55.1)	5 (10.2)	
CA125	<35 U/ml	24 (32.0)	43 (57.3)	8 (10.7)	0.025
	≥35 U/ml	45 (51.7)	32 (36.8)	10 (11.5)	
CEA	<3.4 U/ml	66 (49.6)	52 (39.1)	15 (11.3)	0.0005
	≥3.4 U/ml	3 (11.1)	21 (77.8)	3 (11.1)	
HE4	<105 U/ml	52 (41.6)	63 (50.4)	10 (8.0)	0.0696
	≥105 U/ml	16 (45.7)	12 (34.3)	7 (20.0)	

*Results of *χ* ^2^-test

The relationship between tumor sizes and blood cancer markers was also analyzed. Our results showed that BOT patients with abnormal preoperative CA19-9, CEA and HE4 levels had significantly larger tumor sizes (*P* = 0.0048, *P* < 0.0001, and *P* = 0.0411, respectively) (Table 5).

**Table 5:** Tumor sizes based on categorized blood cancer markers

		Number	Tumor size (cm)	*P* ^*^
CA199	<35 U/ml	99	10.6 ± 6.3	0.0048
	≥35 U/ml	42	14.2 ± 8.1	
CA125	<35 U/ml	64	11.1 ± 6.7	0.4269
	≥35 U/ml	78	12.1 ± 7.3	
CEA	<3.4 U/ml	118	10.5 ± 5.7	<0.0001
	≥3.4 U/ml	22	17.4 ± 9.8	
HE4	<105 U/ml	77	11.0 ± 6.6	0.0411
	≥105 U/ml	33	13.8 ± 8.1	

*Results of *T*-test

## Discussion

Despite a good prognosis, even with recurrence, there has been little consensus concerning optimal management of BOT cases and a lack of clarity on best strategies. Identification of clinicopathological variables predicting recurrence and survival may assist in selection of optimum treatments for BOT patients. This retrospective study showed that certain types of fertility-preserving surgery, lymphadenectomy and invasive implants were associated with PFS of BOT patients. Due to the limited number of deaths, no factors related to OS were identified.

BOT often occurs in younger patients during child-bearing years. Fertility-preserving surgery is an important option for many of these women. Previous studies have failed to reveal an impact on OS of fertility-preserving surgery [6]. The effect of fertility-preserving surgery on recurrence remains inconclusive. Several studies report no impact of fertility-preserving surgery on recurrence [7–9], whereas others report an association with worse PFS [6, 10–14]. Fertility-preserving surgery can be performed in different ways and this can impact outcomes. In comparison to salpingo-oophorectomy, cystectomy retains more normal ovarian tissue and increases rates of pregnancy success [15]. On the other hand, cystectomy may increase the risk of recurrence. Our results showed that PFS was worse for patients that underwent BC compared to patients that underwent radical surgery. There was no significant difference in PFS between the other methods of fertility-preserving surgery (USO and USO + CC) and radical surgery, or among the three styles of fertility-preserving surgery. This is corroborated by findings in other studies: Chen et al. found that the choice of surgical methods used to preserve fertility had significant impact on recurrence and subsequent pregnancy [15]; the same is reported in other studies [16, 17]. Taken together, these results suggest that the types of fertility-preserving surgery have different impacts on oncologic and pregnancy outcomes of BOT patients.

A dilemma encountered in treating BOT patients occurs around the question of whether or not to remove the retroperitoneal lymph nodes during surgery. Shih et al. reported that approximately half of 266 patients underwent lymphadenectomy and that lymphadenectomy was not significantly associated with PFS in their study [9]. Other studies also report that lymphadenectomy did not improve PFS or OS for BOT patients [18–22]. In our study, a total of 99 patients (56.2%) underwent lymphadenectomy and this was significantly associated with improved PFS. This finding is consistent with the results of a previous study [23]. It is also noted that 8.4% of these patients had pelvic lymph node metastasis and 16.7% of patients had para-aortic lymph node metastasis. Ureyen et al. found that positive lymph node metastasis was significantly associated with worse PFS in patients with serous BOT [24]. It is plausible that lymphadenectomy may improve PFS through removal of lymph node metastasis.

Several reports indicate that invasive peritoneal implants are associated with a poor prognosis [14, 25–30]. Consistent with these finding, our study showed that presence of invasive implants was significantly associated with worse PFS in BOT patients. These data suggest that BOT patients with invasive implants may require more aggressive treatments. Serous BOT with invasive implant is considered to be low-grade serous adenocarcinoma and is associated with a significantly worse prognosis [31]. However, this study included only eight BOT patients (4.4%) that had invasive implants. Our data did not reveal a difference in PFS among patients with BOT of different histological types. Conversely, invasive implants may not be identified as a prognostic factor due to inaccuracy of implant diagnosis made for various reasons [32].

Adjuvant chemotherapy has been shown to significantly increase toxicity without therapeutic benefits in BOT patients with stage I disease [33]. Other studies report no benefits of adjuvant chemotherapy regardless of stage or tumor histology [34, 35]. A meta-analysis revealed no significant effect of adjuvant chemotherapy on survival in BOT patients with invasive implants [36]. Findings reported here are in agreement with these reports: our multivariate model showed adjuvant chemotherapy is not a factor that is significantly associated with PFS.

It has been reported that serous BOT is more common in Western countries and mucinous histology is more common in Asian countries [37]. More cases of mucinous vs serous BOT were observed in the Chinese patients in our study (44.9% vs 39.9%). In addition, mucinous tumors (15.2 ± 7.7 cm) were significantly larger than both serous (8.3 ± 3.9 cm) and endometroid tumors (8.6 ± 4.8 cm) (Both *P* < 0.01), as reported previously [38, 39]. However, multivariate analysis performed in this study revealed no significant difference in PFS in patients with serous BOT compared to those with mucinous BOT.

Higher levels of the serum tumor markers CA125 and CA19-9 have been shown to be associated with larger tumor size. The elevation of serum CA125 may suggest serous tumors [40], while high levels of serum CA19-9 and CEA may indicate mucinous BOTs [41]. In our study, we also observed that sizes of BOT were significantly associated with higher levels of CA19-9, CEA and HE4. Significantly more patients with serous BOT had abnormal CA125 (≥35 U/ml), and significantly more patients with mucinous BOT had abnormal CEA levels (≥3.4 U/ml). Although previous studies have suggested that preoperative blood CA125 levels may serve as a prognostic marker for BOT patients [9, 42], our study did not reveal an association between PFS and preoperative blood levels of CA125, CA19-9, CEA or HE4.

The retrospective nature of this study, the relatively small number of patients, and the short follow-up time may present limitations to the application of findings reported here. A small number of patients (5) represented in this study died of the disease. Fertility outcomes of BOT patients that underwent fertility-preserving surgery is not available.

## Conclusions

Our study reveals that certain types of fertility-preserving surgery, lymphadenectomy, and invasive implant are related to PFS in BOT patients. Blood cancer markers may be associated with histology and size of BOT. These findings may assist in selection of optimum management of BOT.

## Additional file

Additional file 1: Table S1. Clinicopathological features of patients with lymphadenectomy. **Table S2.** Clinicopathological features of patients with lymph node metastasis. **Table S3.** Clinicopathological features of patients with chemotherapy. **Table S4.** Information of recurrence sites. **Table S5.** Recurrence outcomes in patients based on clinicopathological classifications. (DOC 92 kb)

## Abbreviations

BC: Bilateral cystectomy
BOTs: Borderline ovarian tumors
FIGO: International Federation of Gynecology and Obstetrics
N/A: Not applicable
OS: Overall survival
PFS: Progressive free survival
USO: Unilateral salpingo-oophorectomy
USO + CC: Unilateral salpingo-oophorectomy plus contralateral cystectomy
