# Supplementary material for: Identification of factors that impact recurrence in patients with borderline ovarian tumors
Source: J Ovarian Res. 2017 Apr 4;10:23. doi: 10.1186/s13048-017-0316-5 (PMC5379723; doi:10.1186/s13048-017-0316-5)
Supplement: Additional file 1: Table S1. — Clinicopathological features of patients with lymphadenectomy. Table S2. Clinicopathological features of patients with lymph node metastasis. Table S3. Clinicopathological features of patients with chemotherapy. Table S4. Information of recurrence sites. Table S5. Recurrence outcomes in patients based on clinicopathological classifications. (DOC 92 kb) [file 13048_2017_316_MOESM1_ESM.doc]

Additional file 1

Table S1. Clinicopathological features of patients with lymphadenectomy

| Variables |  | lymphadenectomy | | P value* |
| --- | --- | --- | --- | --- |
|  |  | No | Yes |  |
| CA125 | <35 U/ml | 42 (53.9) | 36 (46.1) | 0.0114 |
|  | ≥ 35 U/ml | 31 (34.4) | 59 (66.6) |  |
| Paraortic lymph | 0 | 75 (53.6) | 65 (46.4) | <0.0001 |
|  |  | 2 (5.6) | 34 (94.4) |  |
| Recurrence | No | 56 (38.9) | 88 (61.1) | 0.0058 |
|  | Yes | 21 (65.6) | 11 (34.4) |  |

* Results of χ^2^-test

Table S2. Clinicopathological features of patients with lymph node metastasis

| Variables |  | Lymph node metastasis | | P value* |
| --- | --- | --- | --- | --- |
|  |  | No | Yes |  |
| CA125 | <35 U/ml | 35 (97.2) | 1 (2.8) | 0.0102 |
|  | ≥ 35 U/ml | 46 (78.0) | 13 (22.0) |  |
| HE4 | <105 U/ml | 68 (93.2) | 5 (6.8) | <0.0001 |
|  | ≥ 105 U/ml | 12 (57.1) | 9 (42.9) |  |
| Location | Left | 34 (87.2) | 5 (12.8) | 0.0046 |
|  | Right | 36 (94.7) | 2 (5.3) |  |
|  | Bilateral | 14 (63.6) | 8 (36.4) |  |
| Histology | Serous | 30 (68.2) | 14 (31.8) | 0.0002 |
|  | Mucinous | 42 (97.7) | 1 (2.3) |  |
|  | Others | 11 (100) | 0 (90) |  |
| Micropapillary | No | 79 (89.8) | 9 (10.2) | 0.0001 |
|  | Yes | 5 (45.5) | 6 (54.5) |  |
| Invasive implant | No | 83 88.3) | 11 (11.7) | <0.0001 |
|  | Yes | 1 (20.0) | 4 (80.0) |  |
| Stage | 1 | 65 (98.5) | 1 (1.5) | <0.0001 |
|  | 2/3 | 15(53.6) | 13 (46.4) |  |
| Stage | I | 65 (98.5) | 1 (1.5) | <0.0001 |
|  | II/III | 15 (53.6) | 13 (46.4) |  |
| Chemotherapy | No | 77 (91.7) | 7 (8.3) | <0.0001 |
|  | Yes | 7 (46.7) | 8 (53.3) |  |
| Recurrence | No | 77 (87.5) | 11 (12.5) | 0.0374 |
|  | Yes | 7 (63.6) | 4 (36.4) |  |

* Results of χ^2^-test

Table S3. Clinicopathological features of patients with chemotherapy

| Variables |  | Chemotherapy | | P value* |
| --- | --- | --- | --- | --- |
|  |  | No | Yes |  |
| Location | Left | 72 (94.7) | 4 (5.3) | <0.0001 |
|  | Right | 57 (90.5) | 6 (9.5) |  |
|  | Bilateral | 20 (64.5) | 11 (35.5) |  |
| Histology | Serous | 55 (77.5) | 16 (22.5) | 0.0010 |
|  | Mucinous | 76 (95.0) | 4 (5) |  |
|  | Endometrioid | 18 (100) | 0 (0) |  |
| Micropapillary | No | 139 (89.1) | 17 (10.9) | 0.0170 |
|  | Yes | 14 (70.0) | 6 (30.0) |  |
| Invasive implant | No | 151 (89.4) | 18 (10.6) | P<0.0001 |
|  | yes | 3 (37.5) | 5 (62.5) |  |
| Stage | I | 109 (94.8) | 6 (5.2) | <0.0001 |
|  | II/III | 28 (68.3) | 13 (31.7) |  |
| Recurrence | No | 130 (89.7) | 15 (10.3) | 0.0374 |
|  | Yes | 24 (75.0) | 8 (25.0) |  |

* Results of χ^2^-test

Table S4. Information of recurrence sites

| Recurrent sites | N (%) | Lymph node recurrence* | |  |
| --- | --- | --- | --- | --- |
|  |  | Pelvic | Pelvic + Paraaortic |  |
| Same site | 2 (6.3) |  |  |  |
| contralateral site | 14 (43.7) | 1 | 2 |  |
| bilateral sites | 3 (9.4) |  | 1 |  |
| pelvis | 11(34.3) | 1 | 1 |  |
| contralateral site and pelvis | 2 (6.3) |  |  |  |

* Lymph node recurrences were current with recurrences at other sites.

Table S5. Recurrence outcomes in patients based on clinicopathological classifications

| Clinicopathological variables | | Recurrence (N (%)) | | P value* |
| --- | --- | --- | --- | --- |
|  | | No | Yes |  |
| Invasive plant | No | 141 (83.4) | 28 (16.6) | 0.0164 |
|  | Yes | 4 (50.0) | 4 (50.0) |  |
| Lymphadenectomy | No | 56 (72.7) | 21 (27.3) | 0.0058 |
|  | Yes | 88 (88.9) | 11 (11.1) |  |
| Pelvic lymph node metastasis | No | 77 (91.7) | 7 (8.3) | 0.0374 |
|  | Yes | 11 (73.3) | 4 (26.7) |  |
| Tumor size | <10 | 57 (77.0) | 17 (33.0) | 0.0086 |
|  | ≥10 | 72 (92.3) | 6 (7.7) |  |
| Fertility preserving surgery | |  |  | 0.0078 |
| USO | | 27 (81.8) | 6 (18.2) |  |
| USO+CC | | 14 (66.7) | 7 (33.3) |  |
| BC | | 8 (57.1) | 6 (42.9) |  |
| Radical surgery | | 96 (88.1) | 13 (11.9) |  |
| Histology | Serous | 53 (74.7) | 18 (25.3) | 0.0765 |
|  | Mucinous | 71 (88.8) | 9 (11.2) |  |
|  | Endometrioid | 15 (88.3) | 3 (16.7) |  |
| Stages | I | 103 (89.6) | 12 (10.4) | 0.0104 |
|  | II&III | 30 (73.2) | 11 (26.8) |  |
| Chemotherapy | No | 130 (84.4) | 24 (15.6) | 0.0256 |
|  | Yes | 15 (65.2) | 8 (34.8) |  |
| Micropapillary | No | 133 (85.3) | 23 (14.7) | 0.0010 |
|  | Yes | 11 (55.0) | 9 (45.0) |  |

* Results of χ^2^-test
